# The effect of leadership, emotional stability, and expertise marker on swift trust in first aid: a text-vignette study

**DOI:** 10.3389/fpsyg.2025.1600551

**Published:** 2025-10-24

**Authors:** Wilhelm Brodin, Fredrik Fernlund, Erik Prytz

**Affiliations:** ^1^Department of Computer and Information Science, Linköping University, Linköping, Sweden; ^2^Center for Disaster Medicine and Traumatology, Linköping University, Linköping, Sweden

**Keywords:** swift trust, first aid, autocratic leadership, emotional stability, expertise marker

## Abstract

Autocratic leadership and emotional stability have been found to evoke more swift trust in a first aid context. However, it is still unknown how markers of emergency response expertise affect swift trust in this context. The current study aimed to partially replicate the effect of autocratic leadership and emotional stability and investigate the effect of an expertise marker on swift trust in first aid. Swift trust was measured in text vignettes of a first aid scenario with a 2×2 design (autocratic leadership and emotional stability versus democratic leadership and emotional instability, and presence versus absence of emergency response expertise marker). The results show an interaction effect between leadership behavior and emotional stability with the expertise marker. This suggests that people giving first aid while wearing an expertise marker are expected to show clear and direct leadership and emotional stability for increased swift trust. The positive effect of autocratic leadership and emotional stability on swift trust was also replicated. Future work should investigate more diverse first aid scenarios that are found in real-life first aid.

## Introduction

When accidents happen, immediate responders or civil response persons are often first to respond, before a professional emergency response (Bakke et al., 2015; Pilemalm et al., 2013; Ramsell et al., 2017). Immediate responders are simply people who happen to be nearby when the accident occurs and decide to help. A civil response person, on the other hand, is a volunteer that has received rudimentary training in emergency response and who is dispatched to provide initial basic response before the arrival of professional emergency response (Pilemalm et al., 2013; Ramsell et al., 2017). Both types of responders face difficult circumstances as they try to provide aid, particularly when there is more than one responder providing aid. As a group they have no or little previous shared history, may lack sufficient resources, and have likely not trained to perform together (Whittaker et al., 2015; Majchrzak et al., 2007). What little research has been done on first aid groups shows that they sometimes, but not always, provide better aid when working together (Takei et al., 2014; Pelinka et al., 2004).

Swift trust has been proposed to be an important enabler for cooperation in emergent ad-hoc groups in dangerous situations (Olsen, 2018) and has been linked to increased first aid performance in an experimental setting (Brodin et al., 2025). Olsen et al. (2020) investigated factors associated with swift trust in first aid using video vignettes and found that an autocratic leadership style together with emotional stability elicited the highest trust. Affiliation with institutional organizations, i.e., an expertise marker, has also been proposed and found to positively influence swift trust (Meyerson et al., 1996; Blomqvist and Cook, 2018; Barrett, 2025). However, this has not been tested in the context of first aid.

Olsen et al. (2020) used video vignettes to investigate swift trust formation. An alternative method for this type of study would be text vignettes. Text vignettes are more resource effective, as they do not require recording the different scenarios. However, as the majority of the people have never experienced an actual first aid event, they may not be able to visualize a first aid scenario with sufficient accuracy based on text only. To explore if text vignettes are a viable alternative for research in a first aid context, the current study will attempt to replicate the results of Olsen et al. (2020) using text vignettes instead of video vignettes.

The current study aimed to replicate the findings of Olsen et al. (2020) and also to investigate the effect of an expertise marker on swift trust in first aid. Two research questions were investigated in relation to the aim: (1) can the result from Olsen et al. (2020) be replicated with text vignettes? and (2) what effect does a marker of expertise have on trust in first aid?

## Theoretical framework

**Figure 1:**
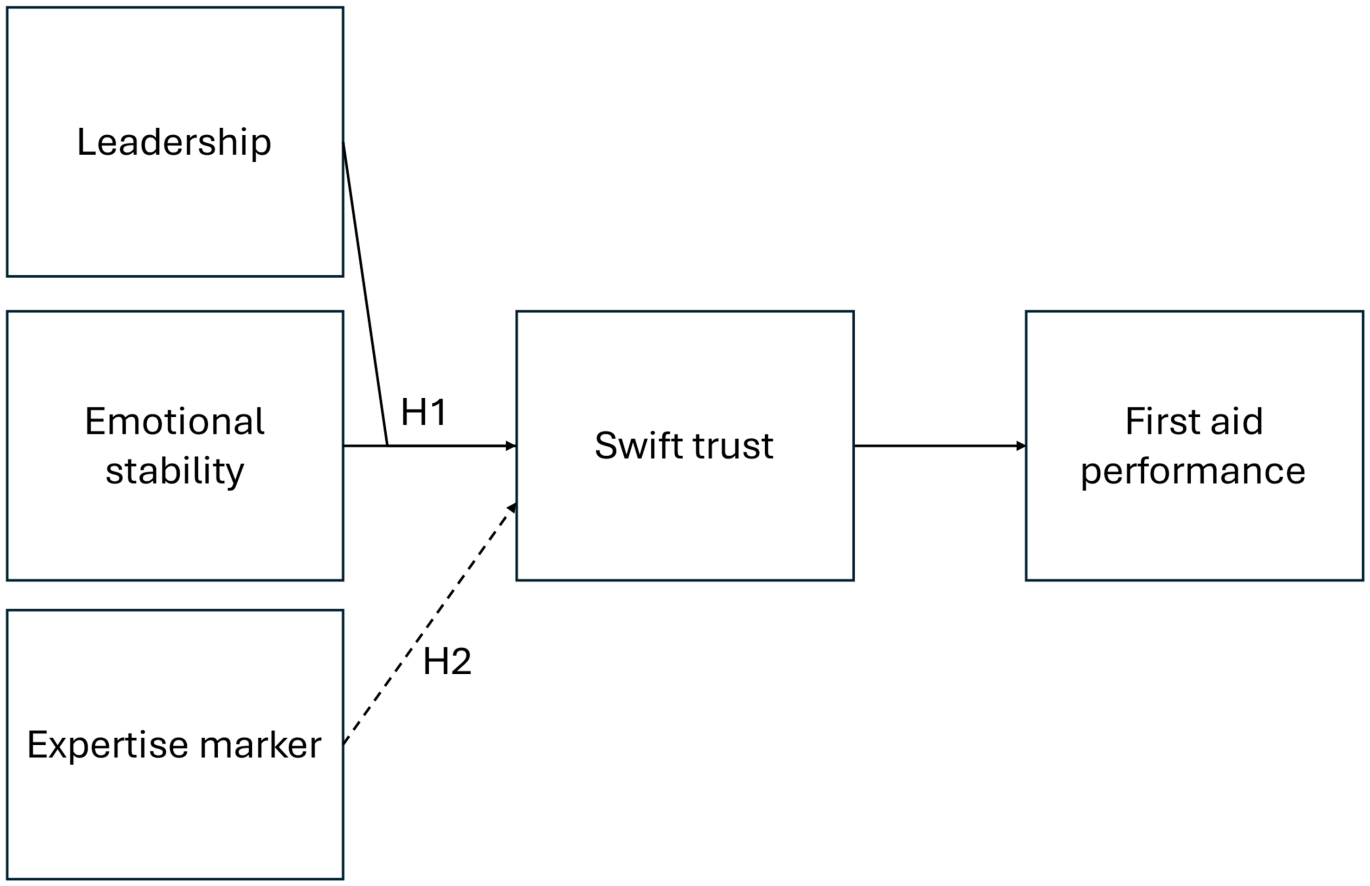
Proposed theoretical framework connecting autocratic and democratic leadership, emotional stability, and expertise marker presence with swift trust and first aid performance. *H1* represents the hypothesis for text vignettes in investigating swift trust in first aid. *H2* represents the hypothesized positive effect of expertise marker presence on swift trust. The dashed line represents the proposed effect of the expertise marker on swift trust with the corresponding hypothesis (*H2*).

Previous research on swift trust and first aid can be constructed into a theoretical framework (see Figure 1). Swift trust is defined by Meyerson et al. (1996) as trust in temporary systems where the decision to trust was made in an instant due to time constraints and lack of previous shared familiarity. Previous research has identified assigning and clearly communicating roles (Kroeger et al., 2021) and brief verbal interaction (Schilke and Huang, 2018) as fundamental to swift trust development and accuracy. Swift trust formation in professional emergency responder teams has been found to be category-based and to rely on third-party recommendations (Xu and Zhao, 2011). In early emergency response by immediate responders (i.e., people giving first aid in emergencies before the arrival of professional responders), swift trust has instead been posited to develop due to the recognition of actions being done expertly (Majchrzak et al., 2007). In other words, swift trust in emergency response by immediate responders may depend on individuals’ active engagement with the shared task (Meyerson et al., 1996; Barrett, 2025) and the apparent membership of organizations (Majchrzak et al., 2007), i.e., institutional categories (Meyerson et al., 1996; Blomqvist and Cook, 2018; Barrett, 2025). The positive effect of organizational membership and institutional categories on swift trust is shown in the framework through a dashed arrow from expert marker to swift trust separately from leadership and emotional stability, as there was little overlap in the first aid literature (see Figure 1). The line is dashed, as the effect is hypothetical in the context of first aid.

Previous research on swift trust in the first aid context has also found autocratic leadership and emotional stability to increase swift trust in a first aid context (Olsen et al., 2020). Olsen et al. conducted a vignette study where participants rated their trust after viewing a short video with an actor displaying autocratic or democratic leadership, and emotional stability or instability. The results showed that the combination of autocratic leadership and emotional stability elicited the largest trust ratings by participants, while democratic leadership and emotional instability elicited the lowest trust (Olsen et al., 2020). Emotional stability is defined in Olsen et al.’s (2020) as an individual’s tendency to slow arousal and fast inhibition (Eysenck and Eysenck, 1985). Low emotional stability, or emotional instability, would then be quick to arousal and have slow inhibition. Autocratic leadership involves taking control of the decision-making and responsibility for the performance of the group or team while democratic leadership asks for input and shares information and decision-making with the group (Bass and Stogdill, 2008; Hannah et al., 2009; Hyllengren et al., 2011). In the framework, the findings of Olsen et al. (2020) that autocratic leadership and emotional stability elicit more swift trust than democratic leadership and emotional instability are visualized by combining the arrows from leadership and emotional stability before reaching swift trust (see Figure 1).

Olsen et al. (2020) used video vignettes to investigate the role of leadership behaviors on swift trust formation in a first aid context. Video vignettes can convey detailed behavior but can be resource-intensive to develop to a sufficient level of quality (van Zelderen et al., 2024). Comparatively, vignettes using text require fewer resources to develop but may lack sufficient detail to immerse the participants in the scenario. The first aid domain is unfamiliar to the majority of people, and the use of text vignettes puts a lot of responsibility on the participant to visualize the described scenario correctly enough for the study’s purpose. It is therefore of interest to test the viability of text vignettes to investigate psychological aspects of first aid research. The hypothesis for RQ1 on using text vignettes instead of video vignettes to investigate swift trust in first aid is thus (see *H1* in Figure 1):

*H1—*The same effect of autocratic leadership and emotional stability eliciting more swift trust compared to democratic leadership and emotional instability can also be found using text-vignettes.

Whereas Olsen et al. (2020) found that leadership and emotional stability influenced swift trust formation, it is unknown how this effect is affected by the presence of an organizational membership or expertise marker (*H2* in Figure 1). One expertise organization in the first aid space currently under development is the civilian response person (Swedish: civil insatsperson, or CIP) initiative (Pilemalm et al., 2013; Ramsell et al., 2017). The CIP initiative aims to identify areas of Sweden that are underserved concerning the emergency response capacity in the region. In these areas, local residents are recruited, educated in basic emergency response, and then included as an early local response capacity to provide basic emergency response before the arrival of professional first responders. These CIP responders are provided with some materials, e.g., a high-visibility reflective vest with “civil insatsperson” written on the back. This vest functions as a marker of both the person’s expertise in emergency response and affiliation with the professional emergency response organization. Thus, the vest functions as an expertise marker since it explicitly communicates the membership of the CIP initiative and emergency response organization (Meyerson et al., 1996; Blomqvist and Cook, 2018; Barrett, 2025). Therefore, the hypothesis for RQ2 regarding the effect of an expertise marker on swift trust in first aid is as follows (see *H2* in Figure 1).

*H2—*The presence of an expertise marker is expected to have a positive effect on swift trust.

## Method

**Figure 2:**
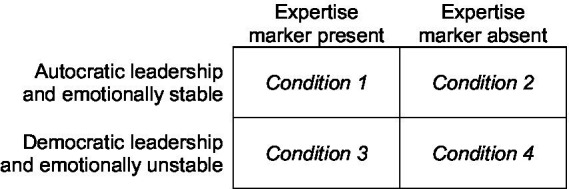
Experimental conditions.

A text-vignette study with a 2 (leadership and emotional stability) by 2 (expertise marker) design was conducted. The two leadership and emotional stability conditions were based on the study by Olsen et al. (2020). Olsen et al. used the separate dimensions of leadership (autocratic versus democratic) and emotional stability (stable versus unstable) for their four conditions. The current study is a partial replication, as only the condition of autocratic leadership combined with emotionally stable behavior and the condition of democratic leadership with emotionally unstable behavior are included. These conditions were selected as they showed the largest differences in reported trust in Olsen et al. (2020). The second dimension added in the current study was the inclusion of a visible expertise marker, see Figure 2 for conditions.

The text vignettes in this study are based on the video vignettes used by Olsen et al. (2020), where each vignette is a textual description of the same scenario and behavior as the corresponding video vignette. An expertise marker was added to this description. The text vignettes were validated by eight emergency response experts to ensure that each condition entailed plausible behavioral descriptions. A convenience sample of 93 participants (43 females, 50 males, and 1 preferred not to answer) with an average age of 28.7 years (SD = 9.9) was then recruited from a university in southern Sweden. Participants received no monetary compensation. The study entailed no risk, danger, collection of sensitive personal data, or physical or psychological manipulation, and thus no prior ethical approval was required according to Swedish law.

### Procedure and materials

First, the participants provided informed consent. They were then distributed into one of four conditions (see Figure 2 for conditions) and answered a question on their propensity to trust based on Delhey et al. (2011), “Would you agree that most people in general can be trusted?”. Participants then read the text vignette and answered three questions about how they thought they would have experienced and acted in the scenario (“I trusted the person”; “I would have followed the person’s instructions”; “I did not trust the person”). Finally, to validate the conditions, the participants answered two questions about how autocratic/democratic and stable/unstable the person in the vignette was perceived (see Table 1 for descriptions of all questions).

**Table 1:** Questions and scales used in the study.

Measurement	Operationalization	Scale	Analysis
Propensity to trust	Would you agree that most people in general can be trusted?	1 (does not agree)—5 (completely agree) Likert scale	Single question
Experience of trust in the scenario	I trusted the person	1 (does not agree at all)—7 (completely agree) Likert scale	Aggregated
	I would have followed the person’s instructions	1 (does not agree at all)—7 (completely agree) Likert scale	Aggregated
	I did not trust the person	1 (does not agree at all)—7 (completely agree) Likert scale	Reverse coded & aggregated
Experience of vignette	I perceived the person as more…	1 (autocratic)—5 (democratic) Likert scale and “Do not know”	Single question
	I perceived the person as more…	1 (unstable)—5 (stable) and “Do not know”	Single question

The text vignettes consisted of a short description of a traffic accident and the interaction between the participant, their friend, and a person who has started to act as an immediate responder. The description of the immediate responder’s behavior and appearance varied between conditions. The following excerpt is the vignette from condition 1, translated from Swedish to English. The first paragraph of the text remained the same for all conditions (see Supplemental material for translations of all vignettes).

*You are out on an evening walk with a friend. It is dark and cold outside. You walk past an industrial area close to a stream when you suddenly sense the smell of gasoline and smoke. When you walk around the corner of the next building you see a car in the middle of the road. Fire is coming from the hood of the car. A man with a high visibility reflective vest is standing a few paces from the car. When he sees you, he shouts “Hey, you two. Can you help me?”*

*You run up to the man and when you get closer you see that it says civil response person on his vest. When you arrive, he says “An accident has happened, a car is on fire and in flames.”*

*He points to the car with his hand “We have to do something.”*

*He points to your friend, “You call emergency dispatch.”*

*He then points to you, “We will make sure to get a better overview and do what’s necessary.”*

*Then he looks at you both, puts his hands together, and says “Good, let us get started.”*

*Text-vignette, condition 1.*

#### Analysis

Two independent sample t-tests were conducted to validate the text-vignettes’ representation of leadership and emotional behaviors. These were grouped according to the leadership and emotional stability dimensions, i.e., comparing conditions 1 and 2 with conditions 3 and 4, to test differences with regard to rated autocratic/democratic behavior and rated emotional stability/instability. Mann–Whitney *U*-test was used as a non-parametric alternative in case of violation of the assumption of normality.

A 2×2 ANCOVA with propensity to trust as a covariate was conducted to investigate if the effect from Olsen et al. (2020) could be replicated using text vignettes as well as the effect of an expertise marker in the behavioral description of the vignette. *Post-hoc* comparisons with Bonferroni corrections were made between every condition. The significance level was set to 0.05 for all analyses.

## Results

The Mann–Whitney *U*-tests for leadership and emotional stability ratings were significant. Leadership was rated significantly different in conditions with autocratic leadership and emotional stability (condition 1 & 2; *N* = 41, *Mdn* = 2) compared to conditions with democratic leadership and emotional instability (condition 3 & 4; *N* = 37, *Mdn* = 4) *U* = 120, *p* < 0.01, *r* = −0.23. Emotional stability was rated differently in conditions with autocratic leadership and emotional stability (conditions 1 & 2; *N* = 44, *Mdn* = 5) compared to conditions with democratic leadership and emotional instability (conditions 3 & 4; *N* = 46, *Mdn* = 2) *U* = 155, *p* < 0.01, *r* = 0.2. This indicates that vignettes 1 and 2 were rated as more autocratic and emotionally stable than vignettes 3 and 4, whereas vignettes 3 and 4 were rated as more democratic and emotionally unstable.

**Figure 3:**
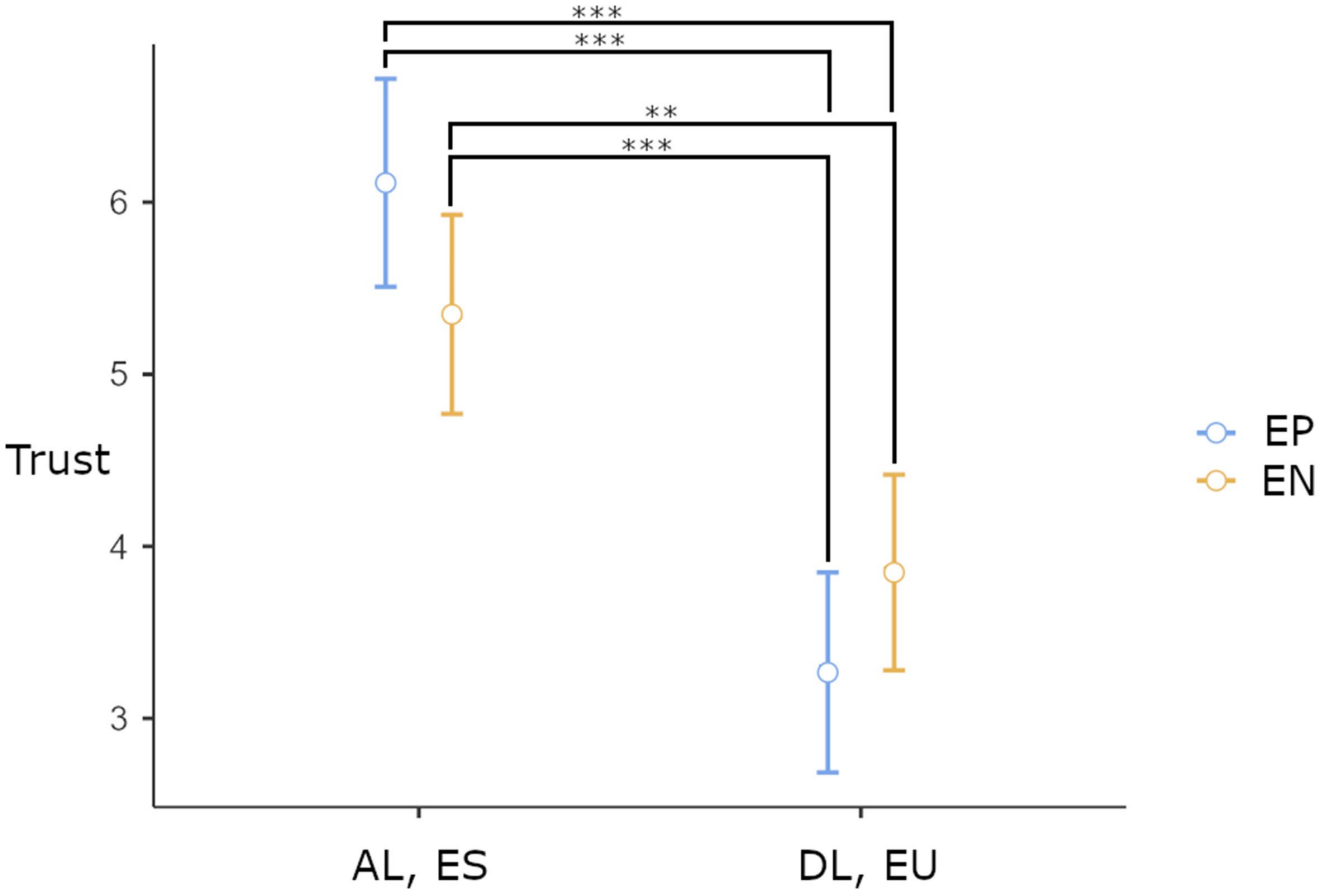
Estimated marginal means with standard errors and *post-hoc* significance results of the ANCOVA. AL, autocratic leadership; ES, emotionally stable; DL, democratic leadership; EU, emotionally unstable; EP, expert marker present; EN, expert marker not present. *p < 0.05, **p < 0.01, ***p < 0.001.

In the 2×2 ANCOVA on the effect of leadership, emotional stability, and expertise marker presence on rated trust, the covariate propensity of trust was found to be significantly related to trust, *F*(1, 88) = 8.4, *p* = 0.005, *r* = 0.23. There was also a significant main effect of leadership behavior and emotional stability on trust *F*(2, 88) = 56.1, *p* < 0.001, partial *η*^2^ = 0.39, and a significant interaction effect between leadership behavior and emotional stability and the presence of an expertise marker, *F*(2,88) = 5.29, *p* = 0.024, partial *η*^2^ = 0.06. A *post-hoc* analysis comparing all four conditions with each other found four significant differences between conditions (see Table 2; Figure 3).

**Table 2:** *Post-hoc* comparisons of experiment conditions.

Comparison		Difference	*t*	*p*	*d*
Condition 1	Condition 2	0.77	1.78	0.47	0.55
	Condition 3	2.85	6.8	<0.001***	2.04
	Condition 4	2.27	5.39	<0.001***	1.62
Condition 2	Condition 3	2.08	5.01	<0.001***	−1.49
	Condition 4	1.5	3.7	0.002**	1.07
Condition 3	Condition 4	−0.58	−1.42	0.96	−0.42

Df = 88. *p < 0.05, **p < 0.01, ***p < 0.001.

## Discussion

### Theoretical implications

RQ1 in the current study aimed to determine if the results from Olsen et al. (2020) could be replicated with text vignettes. The results from the Mann–Whitney *U*-tests showed a difference in both perceived leadership behavior and emotional stability between conditions. This validates the operationalization of autocratic and democratic leadership and emotional stability and instability in text vignettes. The *post-hoc* tests of the ANCOVA also showed significant differences between autocratic, emotionally stable, and democratic emotionally unstable conditions with regard to the participants’ reported trust. Thus, the findings of Olsen et al. (2020) were replicated using text vignettes validated to operationalize the intended behavioral descriptions, and *H1* was confirmed. This, in turn, indicates that text-vignettes are a viable option for investigating psychological aspects of the first aid domain.

The second research question aimed to investigate the effect of an expertise marker on rated trust in first aid. There was a significant interaction effect involving the expertise marker, leadership behavior, and emotional stability. The interaction effect showed the order of highest-rated trust reversed between conditions 1 and 2 compared to conditions 3 and 4. This means that expertise markers increased trust in groups with autocratic leadership behaviors and emotional stability but decreased it in groups with democratic leadership behaviors and emotional instability. Hence, individuals with expertise markers may exhibit expected behavior that does not include either democratic leadership, emotionally unstable behavior, or both. *H2* was thus only supported when behaviors of autocratic leadership and emotional stability were also present. Future work should aim to ascertain which dimension of behavior by individuals with expertise markers should be avoided in scenarios where trust has a high impact on the aid provided, such as in emergent ad-hoc first aid groups (Brodin et al., 2025).

### Limitations

The expert marker used in the current study was a description of a reflective vest with “civil insatsperson” (English: civilian response person), which is a Swedish initiative that is not yet present in all parts of Sweden. Some participants may therefore have been unfamiliar with the meaning of the marker. The unfamiliarity with the CIP initiative may partially explain the lack of main effect on the presence of an expert marker in the ANCOVA. Signifiers of expertise (e.g., symbols or actions) have previously been proposed to increase trust in immediate responder groups (Majchrzak et al., 2007). However, the CIP initiative could be expected to have similar levels of familiarity in the real world, and thus the interaction effect showing a decline in trust in democratic leadership and emotional instability exemplifies behavior that people acting as a CIP may want to avoid in cases where trust is needed. For example, swift trust may play an important part in scenarios where a CIP needs to collaborate with immediate responders (Majchrzak et al., 2007). The swift trust in ad-hoc immediate responder groups is in part based on the appearance of expertise in actions (Majchrzak et al., 2007). The text vignettes were limited to one type of emergency (i.e., car on fire), but other emergencies could be more complex and thus more difficult to show expertise in. Future work should therefore explore trust in different types of emergencies based on immediate responders’ experience of providing first aid in difficult scenarios in real life. For example, the actual needs and priorities in a first aid scenario may be ambivalent and may be the basis for intrapersonal conflicts that could affect trust and the ability to cooperate within the immediate responder group.

### Practical recommendations

Kroeger et al. (2021) showed that clarity of roles and clear communication are some of the most important aspects of swift trust creation. They are studying swift trust in project management, where roles can be assigned *a priori*, but that is not possible in emergent ad-hoc groups responding to a first aid scenario. However, clear communication and role assignment is included in the behavior descriptions in the conditions with autocratic leadership and emotional stabilty, which may explain the increased swift trust in those conditions. First aid educational efforts including aspects of collaboration could include role assignment as a teamwork task for improved swift trust development.

The current study replicates the findings of Olsen et al. (2020) that clear and direct leadership and emotional stability are important for the creation of swift trust in first aid. It also extends this knowledge to the presence of an expertise marker where the results suggest an expectation of autocratic leadership and emotional stability in responders wearing expertise markers for trust to be extended toward them. This further emphasizes the need to include training in leadership and emotional stability in CIP initiatives and democratic leadership and emotional instability as behaviors people acting as CIP may want to avoid. Future work should aim toward extending the findings into more diverse and difficult first aid scenarios.

## Data Availability

The datasets presented in this article are not readily available because of a limitation in the informed consent form. Requests to access the datasets should be directed to wilhelm.brodin@liu.se.

## References

[ref1] Bakke H. K. Steinvik T. Eidissen S. -I. Gilbert M. Wisborg T. (2015). Bystander first aid in trauma—prevalence and quality: a prospective observational study. Acta Anaesthesiol. Scand. 59, 1187–1193. doi: 10.1111/aas.12561 26088860 PMC4744764

[ref2] Barrett A. K. (2025). Swift Trust in Temporary Systems: a systematic review and future research agenda. Small Group Res. doi: 10.1177/10464964251348901

[ref3] Bass B. M. Stogdill R. M. (2008). Bass & Stogdill's handbook of leadership: theory, research, and managerial applications. 4th Edn. New York: Simon and Schuster.

[ref4] Blomqvist K. Cook K. S. (2018). “Swift trust—state-of-the-art and future research directions” in Routledge companion to trust. eds. Searle R. H. Nienaber A. Sitkin S. (London: Routledge), 29–49.

[ref5] Brodin W. Jonson C. O. Johansson M. Prytz E. (2025). Exploring teamwork, trust, and emergency response competence in emergent ad-hoc immediate responder groups: an experimental simulation study. Ergonomics, 1–11. doi: 10.1080/00140139.2025.2478256, PMID: 40111760

[ref6] Delhey J. Newton K. Welzel C. (2011). How general is trust in “most people”? Solving the radius of trust problem. Am. Sociol. Rev. 76, 786–807. doi: 10.1177/0003122411420817

[ref7] Eysenck H. Eysenck M. (1985). Personality and individual differences: a natural science perspective. New York, NY: Springer.

[ref8] Hannah S. T. Uhl-Bien M. Avolio B. J. Cavarretta F. L. (2009). A framework for examining leadership in extreme contexts. Leadersh. Q. 20, 897–919. doi: 10.1016/j.leaqua.2009.09.006

[ref9] Hyllengren P. Larsson G. Fors M. Sjöberg M. Eid J. Kjellevold Olsen O. (2011). Swift trust in leaders in temporary military groups. Team Perform. Manag. 17, 354–368. doi: 10.1108/13527591111182625

[ref10] Kroeger F. Racko G. Burchell B. (2021). How to create trust quickly: a comparative empirical investigation of the bases of swift trust. Camb. J. Econ. 45, 129–150. doi: 10.1093/cje/beaa041

[ref11] Majchrzak A. Jarvenpaa S. L. Hollingshead A. B. (2007). Coordinating expertise among emergent groups responding to disasters. Organ. Sci. 18, 147–161. doi: 10.1287/orsc.1060.0228, PMID: 19642375

[ref12] Meyerson D. Weick K. Kramer R. (1996). Swift trust and temporary groups. I trust in organizations: Frontiers of theory and research. Thousand Oaks: SAGE Publications, Inc.

[ref13] Olsen O. K. (2018). “Effective Cooperation Between Strangers in Unexpected and Dangerous Situations – A Matter of “Swift Trust”. in Interaction: ‘Samhandling’ Under Risk. A Step Ahead of the Unforeseen. ed. G.-E. Torgersen (Oslo: Cappelen Damm Akademisk). doi: 10.23865/noasp.36.ch21

[ref14] Olsen O. K. Heesch P. v. Søreide C. Hystad S. W. (2020). Trust after just 45 seconds? An experimental vignette study of how leader behavior and emotional states influence immediate trust in strangers. Front. Psychol. 10:2921. doi: 10.3389/fpsyg.2019.02921, PMID: 31998185 PMC6962134

[ref15] Pelinka L. E. Thierbach A. R. Reuter S. Mauritz W. (2004). Bystander trauma care—effect of the level of training. Resuscitation 61, 289–296. doi: 10.1016/j.resuscitation.2004.01.012, PMID: 15172707

[ref16] Pilemalm S. Stenberg R. Granberg T. A. (2013). Emergency response in rural areas. Int. J. Inf. Syst. Crisis Response Manag. 5, 19–31. doi: 10.4018/jiscrm.2013040102

[ref17] Ramsell E. Pilemalm S. Andersson Granberg T. (2017). Using volunteers for emergency response in rural areas: network collaboration factors and it support in the case of enhanced neighbors. In The 14th international conference on information systems for crisis response and management, May 21-24, Albi, Occitanie Pyrénées-Méditerranée, France (Vol. 14, pp. 985–995). ISCRAM Association.

[ref18] Schilke O. Huang L. (2018). Worthy of swift trust? How brief interpersonal contact affects trust accuracy. J. Appl. Psychol. 103, 1181–1197. doi: 10.1037/apl0000321, PMID: 29963894

[ref19] Takei Y. Nishi T. Matsubara H. Hashimoto M. Inaba H. (2014). Factors associated with quality of bystander CPR: the presence of multiple rescuers and bystander-initiated CPR without instruction. Resuscitation 85, 492–498. doi: 10.1016/j.resuscitation.2013.12.019, PMID: 24384508

[ref20] van Zelderen A. P. Masters-Waage T. C. Dries N. Menges J. I. Sanchez D. R. (2024). Simulating virtual organizations for research: a comparative empirical evaluation of text-based, video, and virtual reality video vignettes. Organ. Res. Methods. 28, 457–486. doi: 10.1177/10944281241246770

[ref21] Whittaker J. McLennan B. Handmer J. (2015). A review of informal volunteerism in emergencies and disasters: definition, opportunities and challenges. Int. J. Disaster Risk Reduct. 13, 358–368. doi: 10.1016/j.ijdrr.2015.07.010

[ref22] Xu K. Zhao L. (2011). Individual swift trust and cooperation in emergency rescue team members. In IET international conference on smart and sustainable city (ICSSC 2011) (pp. 53–51). Stevenage UK: IET.

