# Supplementary material for: The effect of leadership, emotional stability, and expertise marker on swift trust in first aid: a text-vignette study
Source: Front Psychol. 2025 Oct 24;16:1600551. doi: 10.3389/fpsyg.2025.1600551 (PMC12592126; doi:10.3389/fpsyg.2025.1600551)
Supplement: Supplementary file 1 [file Supplementary_file_1.docx]

# Text-vignettes

#### Autocratic leadership, emotionally stable and expertise marker present

**English**

You are out on an evening walk with a friend. It is dark and cold outside. You walk past an industrial area close to a stream when you suddenly sense the smell of gasoline and smoke. When you walk around the corner of the next building you a car in the middle of the road. Fire is coming from the hood of the car. A man with a high vis vest is standing a few paces from the car. When he sees you he shouts “Hey, you two. Kan you help me?”

You run up to the man and when you get closer you see that it says civil response person on his vest. When you arrive he says “An accident has happened, a car is on fire and in flames”.

He points to the car with his hand “We have to do something”.

He points to your friend, “You call emergency dispatch”.

He then points to you, “We will make sure to get a better overview and do what’s necessary”.

Then he looks at you both, puts his hands together and says “Good, let’s get started”

**Swedish**

Du är ute på en kvällspromenad med en kompis. Det är mörkt ute, och kallt. Ni går förbi ett industriområde i närheten av ett vattendrag när du plötsligt känner lukten av bensin och rök. När du går runt hörnet på nästa byggnad ser du att det står en bil mitt på vägen. Det kommer eldslågor från motorhuven. En man med reflexväst står en bit bort från bilen, när han ser er ropar han, “Hej, ni två där. Kan ni hjälpa mig?”

Ni springer fram mot mannen, när ni kommer närmare ser ni att det står Civil insatsperson på västen. När ni kommer fram berättar han, “Det har skett en olycka, en bil brinner och är övertänd.”

Han pekar med handen mot bilen, “Vi måste göra något.”

Han pekar mot din kompis, “Du ser till att ringa och larma.”

Sedan pekar han mot dig, “Vi två ser till att skaffa översikt och vidtar nödvändiga åtgärder”.

Han tittar på er två och klappar sedan ihop händerna, “Bra, då sätter vi igång.”

#### Autocratic leadership, emotionally stable and expertise marker not present

**English**

You are out on an evening walk with a friend. It is dark and cold outside. You walk past an industrial area close to a stream when you suddenly sense the smell of gasoline and smoke. When you walk around the corner of the next building you a car in the middle of the road. Fire is coming from the hood of the car. A man with a high vis vest is standing a few paces from the car. When he sees you he shouts “Hey, you two. Kan you help me?”

You run up to the man and when you arrive he says “An accident has happened, a car is on fire and in flames”.

He points to the car with his hand “We have to do something”.

He points to your friend, “You call emergency dispatch”.

He then points to you, “We will make sure to get a better overview and do what’s necessary”.

Then he looks at you both, puts his hands together and says “Good, let’s get started”

**Swedish**

Du är ute på en kvällspromenad med en kompis. Det är mörkt ute, och kallt. Ni går förbi ett industriområde i närheten av ett vattendrag när du plötsligt känner lukten av bensin och rök. När du går runt hörnet på nästa byggnad ser du att det står en bil mitt på vägen. Det kommer eldslågor från moturhuven. En man med reflexväst står en bit bort från bilen, när han ser er ropar han, “Hej, ni två där. Kan ni hjälpa mig?”

Ni springer fram mot mannen. När ni kommer fram berättar han, “Det har skett en olycka, en bil brinner och är övertänd.”

Han pekar med handen mot bilen, “Vi måste göra något.”

Han pekar mot din kompis, “Du ser till att ringa och larma.”

Sedan pekar han mot dig, “Vi två ser till att skaffa översikt och vidtar nödvändiga åtgärder”.

Han tittar på er två och klappar sedan ihop händerna, “Bra, då sätter vi igång.”

#### Democratic leadership, emotionally unstable and expertise marker present

**English**

You are out on an evening walk with a friend. It is dark and cold outside. You walk past an industrial area close to a stream when you suddenly sense the smell of gasoline and smoke. When you walk around the corner of the next building you a car in the middle of the road. Fire is coming from the hood of the car. A man with a high vis vest is standing a few paces from the car. When he sees you he shouts “Hey, you two. Kan you help me?”

You run up to the man and when you get closer you see that it says civil response person on his vest. When you arrive he says ”T-there has been an accident, ehhh, a car is on fire and in flames.

He looks back and fourth between you and your friend. “Ehhh we probably should do something, do you have any suggestions?” he then says.

Your friend nods and says, “I can call emergency dispatch”.

The man looks towards you, “Ehh- Then maybe we can get some sort of overview and, well.. like see if something needs to be done, or something like that.”

He looks back and forth between you and your friend again, “is there anything else that we should consider?.. No?.. Then maybe we should get started”.

**Swedish**

Du är ute på en kvällspromenad med en kompis. Det är mörkt ute, och kallt. Ni går förbi ett industriområde i närheten av ett vattendrag när du plötsligt känner lukten av bensin och rök. När du går runt hörnet på nästa byggnad ser du att det står en bil mitt på vägen. Det kommer eldslågor från moturhuven. En man med reflexväst står en bit bort från bilen, när han ser er ropar han, “Hej, ni två där. Kan ni hjälpa mig?”

Ni springer fram mot mannen, när ni kommer närmare ser ni att det står Civil insatsperson på västen. När ni kommer fram berättar han, “D-det har skett en olycka här, ehhh, en bil brinner och är övertänd.”

Han tittar framåt tillbaka mellan dig och din kompis. “Ehh, vi borde nog göra något, har ni några förslag?” säger han sen.

Din kompis nickar och säger, “Jag kan ringa och larma”.

Mannen tittar vidare mot dig, “Öhh- Då kanske vi kan skaffa någon slags översikt och, ja... typ se om det behövs några andra åtgärder, eller nåt sånt.”

Han tittar framåt tillbaka mellan dig och kompisen igen, “Är det något annat som vi borde tänka på?.. Nej?.. Då kanske vi borde köra igång”.

#### Democratic leadership, emotionally unstable and expertise marker not present

**English**

You are out on an evening walk with a friend. It is dark and cold outside. You walk past an industrial area close to a stream when you suddenly sense the smell of gasoline and smoke. When you walk around the corner of the next building you a car in the middle of the road. Fire is coming from the hood of the car. A man with a high vis vest is standing a few paces from the car. When he sees you he shouts “Hey, you two. Kan you help me?”

You run up to the man and when you arrive he says ”T-there has been an accident, ehhh, a car is on fire and in flames.

He looks back and fourth between you and your friend. “Ehhh we probably should do something, do you have any suggestions?” he then says.

Your friend nods and says, “I can call emergency dispatch”.

The man looks towards you, “Ehh- Then maybe we can get some sort of overview and, well.. like see if something needs to be done, or something like that.”

He looks back and forth between you and your friend again, “is there anything else that we should consider?.. No?.. Then maybe we should get started”.

**Swedish**

Du är ute på en kvällspromenad med en kompis. Det är mörkt ute, och kallt. Ni går förbi ett industriområde i närheten av ett vattendrag när du plötsligt känner lukten av bensin och rök. När du går runt hörnet på nästa byggnad ser du att det står en bil mitt på vägen. Det kommer eldslågor från moturhuven. En man med reflexväst står en bit bort från bilen, när han ser er ropar han, “Hej, ni två där. Kan ni hjälpa mig?”

Ni springer fram mot mannen. När ni kommer fram berättar han, “D-det har skett en olycka här, ehhh, en bil brinner och är övertänd.”

Han tittar framåt tillbaka mellan dig och din kompis. “Ehh, vi borde nog göra något, har ni några förslag?” säger han sen.

Din kompis nickar och säger, “Jag kan ringa och larma”.

Mannen tittar vidare mot dig, “Öhh- Då kanske vi kan skaffa någon slags översikt och, ja... typ se om det behövs några andra åtgärder, eller nåt sånt.”

Han tittar framåt tillbaka mellan dig och kompisen igen, “Är det något annat som vi borde tänka på?.. Nej?.. Då kanske vi borde köra igång”.
